# Development pattern of ocular biometric parameters and refractive error in young Chinese adults: a longitudinal study of first-year university students

**DOI:** 10.1186/s12886-022-02440-9

**Published:** 2022-05-14

**Authors:** Xue Bai, Nan Jin, Qingxin Wang, Yicheng Ge, Bei Du, Di Wang, Qiang Su, Biying Wang, Chi-ho To, Ruihua Wei

**Affiliations:** 1 Tianjin Key Laboratory of Retinal Functions and Diseases, Tianjin Branch of National Clinical Research Center for Ocular Disease, Eye Institute and School of Optometry, Tianjin Medical University Eye Hospital, 251 Fukang Road, Nankai District, Tianjin, China; 2 School of Optometry, The Hong Kong Polytechnic University, Hung Hom, Hong Kong, China; 3 Centre for Eye and Vision Research, 17W Hong Kong Science Park, Hong Kong, China

**Keywords:** Young Chinese adults, Ocular biometric parameters, Subjective refraction, Myopia, Risk factors, Playing online games

## Abstract

**Background:**

The increase in the prevalence of myopia has become a matter of serious public health concern, and few studies to date have examined the ocular biometric parameters of myopia in young Chinese adults. This study aimed to investigate the longitudinal ocular biometric and refractive development of first-year university students and the influence of near work.

**Methods:**

This study included 526 first-year university students from Tianjin Medical University (mean age, 18.34 years; 313 females and 213 males). From 2016 to 2018, participants underwent ocular biometry measurements and subjective refraction annually. Near-work activities such as the use of electronic devices, online games, reading, and writing as well as demographic data were recorded by questionnaires.

**Results:**

The prevalence of myopia in this population from 2016 to 2018 was 92.40%, 92.59%, and 92.97%, respectively. Importantly, the prevalence of high myopia increased significantly from 20.91% to 28.33% (*P* < .001). The spherical equivalent refraction was significantly more myopic by approximately − 0.38 D (from − 4.18 ± 2.44 to − 4.56 ± 2.57 D; *P* < .001) during the period. The axial length, central corneal thickness, and lens thickness became significantly different (all *P* < .05), and the axial length significantly increased by 0.12 mm during 2 years (*P* < .001). Using binary logistic regression analysis, the data indicated that spending more time on online games (odds ratio, 2.09; 95% confidence interval, 1.33**–**3.29) could speed up the progression of myopia (*P* < .05).

**Conclusions:**

This study showed that the prevalence of high myopia continued to increase in undergraduate students over 2 years. Baseline myopia correlated with myopic shift, the time spent on online games, and parental myopia were significantly associated with an increase in myopia in these young adult populations.

**Supplementary Information:**

The online version contains supplementary material available at 10.1186/s12886-022-02440-9.

## Background

Myopia has become a public healthcare problem that has attracted worldwide attention, particularly in East Asia [1]. It has been predicted that approximately half of the world’s population will have myopia by 2050, according to the present escalating trend [2]. In the latest Anyang University Students Eye Study, the prevalence of myopia reached 83.2% and that of high myopia reached 11.1% [3]. As myopia develops, the axial length (AL) becomes excessively long, which increases the risk of sight-threatening eye diseases such as cataracts, glaucoma, retinal detachment, and myopic macular degeneration [4]. Therefore, to prevent vision loss due to myopia, it is critical to control the occurrence or progression of myopia, particularly to minimize the high myopia population [5].

Although much effort has been devoted to studying the onset time of myopia in children and adolescents [6–8], limited longitudinal studies have been conducted on the prevalence of myopia among university students in China [9]. Although university students are mostly older than 18 years, changes in refractive error have been observed in previous studies [9–11]. University students are considered at risk of myopia progression due to studying. The purpose of the present study was to investigate changes in ocular biological parameters and refraction in terms of the influence of near–work activity.

## Methods

### Participants and examinations

A total of 648 students were selected using stratified cluster sampling, and 526 students (mean age, 18.34 years; female, 313; male, 213) completed the examinations and questionnaire survey from 2016 to 2018. The exclusion criteria were congenital cataract, corneal stromal scar, recent wearing of corneal plastic lens, nystagmus, and previous history of ocular surgeries. All measurements were performed using an autorefractor (KR8900; Topcon, Tokyo, Japan), a phoropter (VT–10; Topcon), slit-lamp microscopy (YZ5X1; 66 Vision Tech Co., Ltd., Jiangsu Province, China), optical biometry (LS900; LENSTAR, Haag–Streit, Switzerland), and optical coherence tomography (DRI OCT Triton; Topcon). In the third year, a questionnaire was administered that included sex, age, and near activities, such as online games, electronic devices (excluding games), and near reading–writing (excluding online courses and electronic devices). In addition, the visual fatigue score questionnaire [12] has also been used to investigate the effect of myopia in the past 2 years. The age, refractive error, and ocular biometric parameters of the missing follow-up population were not significantly different from those of the follow-up population.

### Definitions

Spherical equivalent refraction (SER) is defined as the sphere power + 1/2 cylinder power. According to the published standard [13] and combined with the baseline SER results, the participants were classified into four groups: group 1, non-myopia group, with SER ≥  - 0.5 D; group 2, mild myopia group, with − 3.0 D ≤ SER <  − 0.50 D; group 3, moderate myopia group, with − 6.0 ≤ SER <  − 3.0 D; group 4, high myopia group, with SER <  − 6.0 D).

The crystalline lens power (P_L_) was calculated using Bennett’s formula [14]:

$${P}_{L}=\frac{-1000\mathrm{n}({\mathrm{S}}_{\mathrm{CV}}+\mathrm{K})}{1000\mathrm{n}-(\mathrm{ACD}+{\mathrm{c}}_{1}\mathrm{T})({\mathrm{S}}_{\mathrm{CV}}+\mathrm{K}) }+\frac{1000\mathrm{n}}{{-\mathrm{c}}_{2}\mathrm{T}+\mathrm{V}}$$

where T is the lens thickness (LT), V is the vitreous depth, *n* = 4/3 of the aqueous and vitreous indices, c_1_ = 0.596, and c_2_ =  − 0.358 as estimated using the Gullstrand-Emsley eye model. The SER was defined as S_CV_ = SE/(1 − 0.014 × SE).

### Statistical analysis

Statistical analysis was conducted using the Statistical Program for Social Sciences version 26.0 (IBM Corp., Armonk, NY, USA). The correlation of the SE refractive errors between the right and left eyes was 0.87 (*P* < 0.001, Pearson correlation test). Therefore, only the refractive error data of the right eye are reported in the following analysis. Differences between sexes were analyzed using an independent sample t-test or chi-square test. The ocular biological parameters of different refractive groups were compared using one-way analysis of variance and the least significant difference t-test. Repeated-measures analysis of variance was used to observe longitudinal variation characteristics during the three measurements. A binary logistic regression analysis was conducted to test the risk factors associated with AL elongation during the study period. Differences were considered statistically significant at *P* < 0.05.

## Results

### Change of refraction

In the follow-up population included in the study, the prevalence of myopia in the first year was 92.40% (486), whereas the prevalence of high myopia was 20.91% (110). The prevalence rates of hyperopia and emmetropia were 1.33% (7) and 6.27% (33) respectively. After 2 years, the prevalence of myopia was similar to that in the baseline data (92.40% vs. 92.59% vs.92.97%). However, the prevalence of high myopia increased significantly from 20.91% to 28.33%.

The SER was substantially reduced by − 0.38 D (from − 4.18 ± 2.44 D to − 4.56 ± 2.57 D; F = 41.956; *P* < 0.001), and there was no statistical difference in the change of diopter of cylinder (F = 0.058; *P* > 0.05). There were no significant differences in the change in SER between the male (− 0.32 ± 0.66 D) and female students (− 0.42 ± 0.66 D) (*P* > 0.05). The change in SER in groups 1, 2, 3, and 4 was − 0.28 ± 0.49, − 0.36 ± 0.75, − 0.42 ± 0.51, and − 0.37 ± 0.86 D, respectively. There was no significant difference in the change in SER among the four groups (F = 0.703; *P* > 0.05).

### Changes in ocular biometric parameters

The characteristics and changes in ocular biological parameters of different sexes are shown in Table 1. Except for the change in anterior chamber depth (△ACD) and change in corneal radius (△CR), the 2-year changes in other parameters were significant (*P* < 0.001). The AL significantly increased by 0.12 ± 0.18 mm within 2 years (*P* < 0.001); the central corneal thickness (CCT) slightly decreased by 2.06 μm (*P* < 0.01); the ACD and lens power did not change significantly in 2 years (*P* > 0.05); the LT was significantly thickened approximately 0.04 mm (*P* < 0.01). Comparison of the groups with different types of refractive errors showed no statistical difference in the changes in CCT, ACD, LT, and CR in the different myopia groups (all *P* > 0.05). However, the change in AL (△AL) was greater in the moderate and the high myopia groups (groups 3 and 4) than in the non-myopia and mild myopia groups (groups 1 and 2) (0.07 ± 0.27 vs. 0.09 ± 0.19 vs. 0.14 ± 0.16 vs. 0.14 ± 0.15 mm; *P* < 0.05; Table 2). Only the change in AL was statistically different between the two sexes. Within 2 years, the △AL was 0.14 ± 0.18 mm in females and 0.09 ± 0.17 mm in males. There were no significant differences between the groups (*P* > 0.05).

**Table 1:** Characteristics and changes of ocular parameters in different genders

	**All**	**M**	**F**	***P*-value**
Baseline				
SER	-4.18 ± 2.44	-4.09 ± 2.60	-4.23 ± 2.33	> 0.05
AL	25.17 ± 1.21	25.45 ± 1.21	24.98 ± 1.17	< 0.001
CCT	538.34 ± 32.70	543.57 ± 32.70	534.78 ± 32.27	< 0.01
ACD	3.18 ± 0.23	3.20 ± 0.24	3.16 ± 0.23	> 0.05
LT	3.46 ± 0.21	3.46 ± 0.25	3.46 ± 0.18	> 0.05
CR	7.80 ± 0.25	7.86 ± 0.24	7.76 ± 0.25	< 0.001
Lens power	22.74 ± 1.66	22.19 ± 1.54	23.12 ± 1.64	< 0.001
After 2 years				
SER	-4.56 ± 2.58	-4.42 ± 2.84	-4.65 ± 2.38	> 0.05
AL	25.29 ± 1.23	25.54 ± 1.25	25.12 ± 1.20	< 0.001
CCT	536.27 ± 34.47	541.29 ± 35.15	532.86 ± 33.62	< 0.01
ACD	3.18 ± 0.25	3.22 ± 0.25	3.16 ± 0.23	< 0.01
LT	3.50 ± 0.27	3.49 ± 0.27	3.51 ± 0.27	> 0.05
CR	7.80 ± 0.25	7.86 ± 0.24	7.75 ± 0.24	< 0.001
Lens power	22.76 ± 1.60	22.27 ± 1.51	23.12 ± 1.58	< 0.001
Difference				
△SER	-0.38 ± 0.66**	-0.32 ± 0.66**	-0.42 ± 0.66**	> 0.05
△AL	0.12 ± 0.18**	0.09 ± 0.17**	0.14 ± 0.18**	< 0.01
△CCT	-2.06 ± 15.11**	-2.28 ± 15.35*	-1.91 ± 14.97*	> 0.05
△ACD	0.01 ± 0.10	0.01 ± 0.11	0.00 ± 0.09	> 0.05
△LT	0.04 ± 0.23**	0.02 ± 0.25	0.06 ± 0.22**	> 0.05
△CR	0.01 ± 0.08	0.00 ± 0.06	0.00 ± 0.09	> 0.05
△lens power	0.04 ± 0.92	0.11 ± 0.84	0.01 ± 0.98	> 0.05

The difference △ value represents in change of parameters within two years, *P value means that the test level has a statistical difference at 0.05, and **P value means that the test level has a statistical difference at 0.01

**Table 2:** Changes of ocular parameters in different groups

	**non-myopia**	**mild myopia**	**moderate myopia**	**high** **myopia**	***p* value**
△AL	0.07 ± 0.27	0.09 ± 0.19	0.14 ± 0.16	0.14 ± 0.15	< 0.05
△CCT	-0.90 ± 17.09	-1.08 ± 9.13	-2.21 ± 17.63	-3.33 ± 14.02	> 0.05
△ACD	0.04 ± 0.13	0.02 ± 0.11	0.00 ± 0.09	-0.01 ± 0.08	< 0.01
△LT	0.05 ± 0.28	0.06 ± 0.27	0.03 ± 0.18	0.04 ± 0.28	> 0.05
△CR	0.00 ± 0.08	-0.01 ± 0.06	0.00 ± 0.05	0.01 ± 0.14	> 0.05

The △ value represents the difference in change of parameters within two years

### Correlation between ocular biometric parameters and refraction

**Fig. 1:**
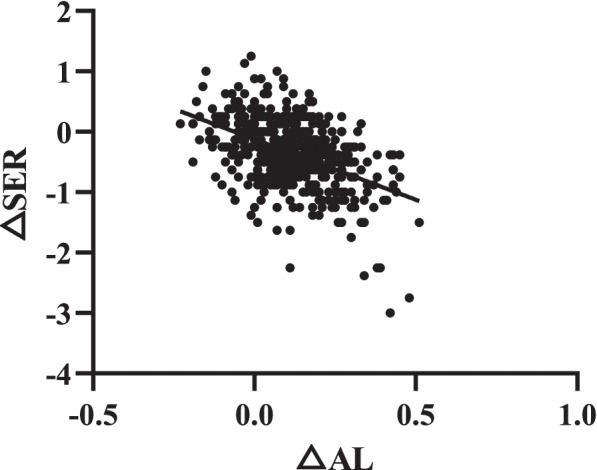
Correlation between △AL and △SER. The △ value represents the difference in change of parameters within two years. AL = axial length, SER = spherical equivalent refraction

A simple linear regression analysis of △AL was performed, and the change in SER (△SER) was obtained as △SER =  -  2.079 × △AL -  0.12 (R^2^ = 0.252; *P* < 0.001), and a scatter plot is shown in Fig. 1. SER was negatively correlated with AL, SER, and ACD (all *P* < 0.001). AL presented a significantly positive correlation with ACD and CR and a negative correlation with LT (all *P* < 0.001; Table 3). In addition to CCT, other ocular parameters showed a significant correlation with SER (AL, *β* =  -  2.23; ACD, *β* = 1.27; LT, *β* =  -  1.84; CR, *β* = 5.52).

**Table 3:** Correlation among ocular biometric parameters

	**AL**	**CCT**	**ACD**	**LT**	**CR**
SER	-0.778**	+ 0.102**	-0.326**	+ 0.138**	+ 0.132**
AL		+ 0.020	+ 0.453**	-0.281**	+ 0.393**
CCT			-0.108**	+ 0.051	+ 0.169**
ACD				-0.369**	-0.040
LT					+ 0.018

^**^P value means that the test level has a statistical difference at 0.01

### Risk factors related to refraction changes

In the binary logistic regression analysis, being female (odds ratio [OR], 2.00; 95% confidence interval [CI], 1.02–3.95), playing online games (OR, 2.09; 95% CI, 1.33–3.29), having one parent with myopia (OR, 1.997; 95% CI, 1.264–3.094), and having one parent with high myopia (OR, 2.183; 95% CI, 1.379–3.441) were significant risk factors for AL elongation. Age, visual fatigue, near reading–writing time (excluding electronic devices), and initial AL were not associated with changes in the AL (Table 4).

**Table 4:** Risk factors for myopic axial eye growth (△AL)

Factor	Β coefficient	OR(95%CI)	P value
Gender	0.694 ± 0.347	2.00(1.02–3.95)	< 0.05
Age	-0.180 ± 0.150	0.84(0.62–1.12)	> 0.05
AL (baseline)	0.260 ± 0.134	1.30(0.99–1.69)	> 0.05
Electronic devices (excluding gaming)	-0.256 ± 0.187	0.77(0.54–1.11)	> 0.05
Online games	0.739 ± 0.231	2.09(1.33–3.29)	< 0.001
Near reading-writing (excluding electronic devices)	0.201 ± 0.322	1.22(0.65–2.30)	> 0.05
Parental myopia	0.682 ± 0.228	1.98(1.26–3.09)	< 0.01
Parents with high myopia	0.779 ± 0.192	2.18 (1.38–3.44)	< 0.01
Visual fatigue score	-0.011 ± 0.021	0.99(0.95–1.03)	> 0.05

## Discussion

### Refraction and prevalence of myopia

To our knowledge, only one longitudinal study [9] has investigated the refraction and ocular biometric parameters of university students in China. In our study, we found that both the severity of myopia and the prevalence of high myopia were significantly increased. Similar observations have been reported in previous studies. During the 5-year follow-up of 345 medical students in Taiwan in 1999, the prevalence of myopia increased from 92.8% to 95.8% [9]. A study of 156 Caucasian medical students in Copenhagen also showed that the prevalence of myopia significantly increased from 37 to 43% over 2 years [15]. In our current population, the overall prevalence of myopia did not change significantly within 2 years (92.40% vs. 92.97%), but the severity of myopia was significantly changed in 2 years: both mild myopia (24.91% vs. 20.34%) and moderate myopia (46.58% vs. 44.30%) decreased, and there was a significant increase in the prevalence of high myopia (20.91% vs. 28.33%). Apparently, mild and moderate myopia may have gradually turned into high myopia in 2 years, which led to an increase in the prevalence of high myopia. Previous studies have researched the average prevalence for all populations with myopia but rarely compared the longitudinal changes in these groups of myopia prevalence separately. The present results of stratifying myopia groups into different severities revealed the gradual migration of mild and moderate myopia into the high myopia group. Control of myopia progression is an important issue, even at this late stage. Prevention of nearly 8% of high myopia cases has a significant public health benefit. This measure should be applied to all medical students and perhaps other university students with myopia. Therefore, to control the high myopia population in university students and prevent subsequent myopia-related vision loss, it is important to slow the escalation of mild/moderate myopia into high myopia. Meanwhile, the cylindrical diopter did not change significantly, which indicated that the astigmatism value was relatively stable in the group of university students and that the changes in myopia or hyperopia were the main reasons for its refractive development.

In this study, the refractive changes during university were similar in the different refractive groups. This showed that refraction was not stable and continued to change, even in late adolescence and early adulthood. This observation may have significant clinical implications for the suitability of refractive laser surgery in this population.

### Ocular biometric parameters

In this study, AL showed a significant increase, especially in female participants. This result was consistent with that of an observational study of medical students in Copenhagen [15]. We speculated that this difference in AL growth may be due to sex difference in stages and rate of growth, and females tend to spend more time studying and less time on outdoor activities. Table 1 describes the change characteristics of ocular biological parameters in different sexes. Previous researches showed that the rate of myopic shift in refraction increases as myopia develops, but it usually increased before children aged ≤ 17 years, and the major determinant of the rate of progression was age, with little dependence on the level of myopia [16–19]. The present study found that the myopic participants showed a significantly faster change in △AL towards myopia than emmetropic participants at baseline. This finding was in accordance with other myopia studies for adults [9, 20, 21]. Further, the present study indicated that participants with moderate myopia and high myopia underwent the fastest change in AL, followed by those with mild myopia and with non-myopia. According to our results of relevant risk factors in the questionnaire, we concluded that there may be two possibilities why does there appear to be a dependence on the level at this late developmental stage. Those that reach to highest levels of myopia may have done so because they have adopted extremely myogenic patterns of behavior, or because they have high genetic risk scores, which may also lead to longer progression at a higher rate.

In this study, there was no significant change in ACD. However, the LT increased significantly by 0.04 mm, and the lens power had no significant change. A similar observation was reported in a cohort study of Portuguese and Norwegian university students [10, 11]. In its 3-year follow-up observation, the LT increased by approximately 0.06 mm in the Portuguese study [11] and 0.07 mm in the Norwegian study [10]. The changes in ACD and LT in the present study were different from previous observations in adolescents. In adolescents, deepening of the ACD and thinning of the LT were observed, which act to compensate for AL elongation. However, no such compensatory changes were observed in the university student population. The stability of ACD and thickening of the LT may have maintained a stable lens power. However, this change was different from the eye development in adolescence and cannot compensate for the change in refraction caused by an increase in AL. We used Bennett’s formula to calculate the lens power in our study, and the results showed the lens power in adolescents with mean age of 18.34 years comes to a plateau (with a decrease of only 0.04 D). This reduction rate was similar to that in adults aged 20 to 50 years who exhibited a monotonous decrease in lens power of approximately 0.37 to 0.12 D every 5 years [22]. We continue to conduct follow-up studies, and we hope to obtain a group of students for the entire period in the future.

### Relevant risk factors in the questionnaire

The search for risk factors for myopia development in adolescents has been intense. The results of previous studies investigating the association between myopia progression and near work are conflicting. Many studies have suggested that near work is an independent risk factor for myopia [23, 24]. A Singapore study [23] showed that children who spent more than 20.5 h reading and writing at a close distance every week were more likely to develop myopia (OR, 1.12). The Sydney myopia study [24] analyzed the near activities of 12-year-old Australian children. The data showed that when reading continuously for more than 30 min, the likelihood of developing myopia was 1.5 times higher than that of controls. A similar result was also reported in a Dutch cohort study [25] on the risk factors of myopia in children aged 6–9 years, but it showed that the total time spent reading per week was not significantly correlated with the occurrence of myopia. Both the medical student cohort study in Copenhagen [15] and the engineering student cohort study in Norway [26] showed that the time spent reading scientific literature was significantly related to the development of myopia. In the present study, the regression analysis results showed that the total daily near reading–writing hours did not correlate with myopic shift (amount of AL growth). This result was similar to that of previous myopia studies on adolescents [25–27], but different from those reported in Beijing students [28, 29]. Because of the different age ranges of the study populations, the total time spent near work had a different effect on myopia in adults than in children.

The use of electronic devices has always been a topic of concern in myopia control. In a Dutch survey, the occurrence of myopia was found to have a significant correlation with the time spent watching television, but not when using the computer [25]. However, in a survey of university students in Norway, the time spent on video display terminals and watching television had no significant effect on refractive changes [26]. In our study, we found that using daily electronic devices (excluding games) had no significant correlation with myopia progression, whereas playing online games had a strong and significant correlation with myopia progression. In particular, the longer the time spent on online games, the greater was the AL elongation (OR, 2.094). Playing online games is frequently a continuous activity that may impose heavy vision stress [24]. In addition, reading–writing is performed with relatively stable light levels and large words compared with computer use, which is related to flashing images; thus, they may have different effects on the eyes. The popular use of electronic screen devices is unlikely to be the culprit of our myopia epidemic in East Asia because the prevalence began much earlier (in the 1980s) than the advent of smartphones and electronic devices [30]. However, the heavy use of these electronic devices would likely increase indoor time, thus further reducing outdoor activities [31].

Among all demographic data collected in this study, only sex and parental myopia were found to correlate with AL changes. In particular, females appear to have a greater risk of developing myopia, as found in several previous studies [3, 32–34]. It is plausible that females tend to spend more time studying, reading, and practicing near work and less time on outdoor activities [26, 35, 36]. In addition, there may be fundamental differences in both the stages and rates of growth between sexes. Similar to a previous Turkish study on medical students [37], parental myopia or high myopia in one of the parents was associated with increased AL. Indeed, parental myopia is an independent risk factor for myopia in young adult populations (OR, 3.69) [37] as it is in adolescents [25]. Intuitively, parental myopia could have genetic and lifestyle roles in influencing the development of myopia in offspring.

This study has some limitations. Only non-cycloplegic refraction data were obtained from university students, and it has the risk of overestimating the prevalence and magnitude of myopia [17]. For greater precision, we suggest that future studies of refractive changes in young adults use the gold standard method of cycloplegic refraction. We did not collect information on the time spent outdoors. A longer time spent outdoors is an effective means of preventing myopia [38–40]. We recommend further research into the effect of outdoor exercise time on myopia in university students. This study was conducted for only 2 years, and it would have been better if we had conducted it for 4 years. In the future, we will continue to follow-up and start a study on cycloplegia.

## Conclusions

The prevalence of high myopia has increased in Chinese university students over the past 2 years. This increase in prevalence is associated with time spent playing online games and parental myopia. The current study highlights the fact that the refraction of our young adult population is not stable but progresses significantly toward high myopia. Control of myopia progression is an important issue, even at this late stage. Therefore, university students are equally in need of myopia control as our adolescent population.

## Supplementary Information

**Additional file 1.** Ocular biometry measurements results.

**Additional file 2.** Questionnaire.

**Additional file 3.** Questionnaires results.

**Additional file 4.** Refraction results.

## Data Availability

The date that supports the findings of this study are available in the supplementary material of this article.
