# Supplementary material for: Development pattern of ocular biometric parameters and refractive error in young Chinese adults: a longitudinal study of first-year university students
Source: BMC Ophthalmol. 2022 May 14;22:220. doi: 10.1186/s12886-022-02440-9 (PMC9107769; doi:10.1186/s12886-022-02440-9)
Supplement: Supplementary file 2 — Additional file 2. Questionnaire. [file 12886_2022_2440_MOESM2_ESM.docx]

Questionnaire for Students of Tianjin Medical University

| Name： | Sex： | Date of birth： | Faculty & Classes： |
| --- | --- | --- | --- |

**1. Which type of correction do you usually use to improve your vision?**

**A. Framing lenses**

**B. Contact lenses (soft lenses)**

**C. RGP (rigid corneal contact lenses)**

**D. Orthokeratology (OK lenses)**

**E. Chinese medicine or visual training etc.**

**F. Nothing**

**2. Have you worn rigid corneal contact lenses in the last month? (OK lens/RGP) Yes□ No□**

**3. Have you had laser surgery for myopia? Yes□ No□**

**4. How do you wear your frames?**

**A. All day B. Only when looking away (e.g. in class) C. Never**

**5. In the two years after school, what is the average daily time spent using near electronic devices (not games) such as mobile phones and computers?**

**A. 0-1h B. 1-2h C. 2-3h D. 3h or more**

**6. Have you played video games (e.g. Jedi, League of Legends, etc.) during the two years after you enrolled in school? Yes□ No□ No**

**If you play video games, how long do you spend on average on video games each day?**

**A. 0-1h B. 1-2h C. 2-3h D. 3h or more**

**7. Do you often work close to each other (e.g. study/read other books) except for daily classes Yes□ No□**

**If you often read close up, what is the average daily reading time?**

**A. 0-1h B. 1-2h C. 2-3h D. 3h or more**

**8. Do you have the habit of buying your own eye drops to dispense?**

**Yes□ No□**

**9. How do you usually buy eye drops?**

**A. hospital B. pharmacy C. online D. overseas (e.g. bought on behalf of others)**

**10. what type of eye drops do you usually buy?**

**A. Artificial tears B. Anti-fatigue C. Antibiotics D. Don't know (please write the name)**

**11. Do either of your parents have myopia?**

**A. Yes B. No C. Don't know**

**12. Is one of the parents highly myopic (>600 degrees of myopia)?**

**A. Yes B. No C. Don't know**

**Visual Fatigue Score Questionnaire**

**Notes:**

1. **First, the frequency, that is, how often the symptom occurs, considering that:**

**NEVER = the symptom does not occur at all**

**OCCASIONALLY = sporadic episodes or once a week**

**OFTEN OR ALWAYS = 2 or 3 times a week or almost every day**

1. **Second, the intensity of the symptom:**

**Remember: if you indicated NEVER for frequency, you should not mark anything for intensity.**

|  | **Frecuency** | | | **Intensity** | |
| --- | --- | --- | --- | --- | --- |
|  | **Never** | **Occasionally** | **Often or always** | **Moderate** | **Intense** |
| **1.Burning** |  |  |  |  |  |
| **2.Itchy** |  |  |  |  |  |
| **3.Feeling of a foreign body** |  |  |  |  |  |
| **4.Tearing** |  |  |  |  |  |
| **5.Excessive blinking** |  |  |  |  |  |
| **6.Eye redness** |  |  |  |  |  |
| **7.Eye pain** |  |  |  |  |  |
| **8.Heavy eyelids** |  |  |  |  |  |
| **9.Dryness** |  |  |  |  |  |
| **10.Blurred vision** |  |  |  |  |  |
| **11.Double vision** |  |  |  |  |  |
| **12.Difficulty focusing for near vision** |  |  |  |  |  |
| **13.Increased sensitivity to light** |  |  |  |  |  |
| **14.Coloured halos around objects** |  |  |  |  |  |
| **15.Feeling that sight is worsening** |  |  |  |  |  |
| **16.Headache** |  |  |  |  |  |

**Calculation of TOTAL SCORE Apply the following expression:**

**Considering that:**

**Frequency:**

**-Never=0**

**-Occasionally=1**

**-Often or always=2**

**Intensity**

**-Moderate=1**

**-Intense=2**
